# Regional Variation in Urinary *Escherichia coli* Resistance Among Outpatients in Washington State, 2013–2019

**DOI:** 10.3390/microorganisms12112313

**Published:** 2024-11-14

**Authors:** Hannah T. Fenelon, Stephen E. Hawes, Hema Kapoor, Ann E. Salm, Jeff Radcliff, Peter M. Rabinowitz

**Affiliations:** 1 Center for One Health Research, Department of Environmental and Occupational Health Sciences, University of Washington, Seattle, WA 98195, USA; 2 Department of Epidemiology, University of Washington, Seattle, WA 98195, USA; 3 Quest Diagnostics, Secaucus, NJ 07094, USA

**Keywords:** antimicrobial resistance, *E. coli*, antibiogram, public health, antimicrobial stewardship

## Abstract

*Escherichia coli* (*E. coli*) is a predominant pathogen of urinary tract infections (UTIs) in the United States. We analyzed resistance patterns by geographic location in Washington State to assess the need for regional antibiograms. The study included urinary *E. coli* antibiotic susceptibility tests performed by Quest Diagnostics on Washington outpatient isolates from 2013 to 2019. We conducted logistic regressions with robust standard errors for five antibiotics (ceftriaxone, ciprofloxacin, gentamicin, trimethoprim-sulfamethoxazole), with isolates classified as “susceptible” or “resistant” for each antibiotic tested. Analyses were adjusted for sex, year of isolate collection, and age group (0–18, 19–50, >50). The state’s nine Public Health Emergency Preparedness Regions (PHEPRs) were used as the geographic level for the analysis. The analysis included 40,217 isolates (93% from females, mean age 47 years). Compared to the Central PHEPR (containing Seattle), most other regions had significantly lower adjusted prevalence ratios (aPORs) of antimicrobial resistance (AMR), with aPORs as low as 0.20 (95% CI: 0.06–0.63) for ceftriaxone in the North Central region. Additionally, no regions had significantly higher aPOR of resistance for any antibiotic. Differences in resistance between the Central and other regions varied by antibiotic with the largest difference for ceftriaxone and smallest for ampicillin. The finding of regional variation of *E. coli* AMR calls for more specific community antibiograms to enable a precise approach to antibiotic prescribing and stewardship.

## 1. Introduction

The global health threat of antimicrobial resistance is growing in scale and severity. The World Health Organization estimates that 10 million people globally will die each year due to antibiotic resistance by 2050 [1]. Bacteria are considered resistant to an antibiotic when the minimum inhibitory concentration threshold set by organizations such as the Clinical and Laboratory Standards Institute is met, meaning it takes more concentrated antibiotics (or a switch to a different antibiotic) to treat an infection by either inhibiting bacterial growth or causing bacterial cell death [2].

Antimicrobial resistance is a concern when treating *Escherichia coli* (*E. coli*), a common uropathogenic bacteria that causes urinary tract infections (UTIs) in the United States. One of the most common bacterial infections in the United States, *E. coli* totals an estimated $5 billion in direct and indirect costs annually [3]. *E. coli* is implicated in 75–95% of uncomplicated cases of cystitis and pyelonephritis, referred to as UTIs, in the United States [4]. Up to 50% of females experience a UTI in their lifetimes [5], and 20–30% develop recurrent UTIs within months of the first infection [3,6]. The prevalence and recurrent nature of UTIs create opportunities for resistance among *E. coli* and highlight the importance of monitoring and preventing antimicrobial resistance in common infections. Better understanding of *E. coli* resistance patterns in UTIs, including risk factors for resistance, will facilitate more timely and effective treatment.

Antimicrobial resistance can be an intrinsic resistance or transmitted vertically, through cell division, and horizontally, through the transfer of genes between bacteria. These antimicrobial resistance genes arise through random advantageous mutations in their genome. There is a spatial component to the reproduction and transference of AMR genes wherein proximity is necessary to transfer resistance promoting genes, and the environmental conditions necessary to make the antimicrobial resistance genes confer a survival benefit and drive population dynamics exist locally [7,8]. Geographic variations in antibiotic prescribing and use patterns may be caused by variations in access to care, provider awareness, and community standards of care, including hospital guidance documents and regional standards [9,10]. Furthermore, anthropogenic impacts on the environment, such as contamination of water and soil with antibiotic residues from antibiotic applications in orchards and on animal farm operations, could influence the evolution of the bacterial antimicrobial resistance genome in the environment and pose a risk to humans in the area [7,11,12]. Abiotic factors, such as the composition of soils, are also associated with variations in antimicrobial resistance persistence in environments [13,14,15], demonstrating the importance of geography and the local environment in understanding the distribution of antimicrobial resistance.

While studies have discovered spatial and temporal associations between antimicrobial resistance in the environment and inpatient settings [16,17], addressing antimicrobial resistance through a spatial lens in an outpatient setting is less researched. Antibiograms are a tool used by providers to aid in appropriate antibiotic selection for their patients by providing aggregate summaries of resistance patterns in a specific setting, such as a hospital or geographic area. While these tools exist, studies have found a gap in the utilization of antibiograms by prescribers, including in a study conducted among American physicians where 70% of the providers surveyed reported access to an antibiogram yet only 50% reported using an antibiogram “always” or “most of the time” [18]. While there is a gap between availability and use, another study found that implementing a frequently updated and unit-specific antibiogram into the electronic medical records system within a hospital saw a steady increase in access of new online antibiograms over the year-long study, showing that availability of an easily accessible and up-to-date antibiogram may be helping staff at the hospital with their prescription decision making [19]. Educating prescribers, making antibiograms more accessible, and creating more accurate antibiograms could help tailor the practice of antibiotic stewardship by prescribers. Should there be significant spatial patterns of resistance, local antibiograms could be created to provide the most regionally accurate information to prescribers. 

The ever-evolving situation surrounding antimicrobial resistance calls for more specific and timely additional guidance for healthcare providers to improve health outcomes and mitigate further resistance to clinically important antibiotics. To further assess the need for localized antibiograms to provide more impactful guidance to practitioners, we assessed regional variations in antimicrobial resistance among outpatient urinary *E. coli* isolates from Washington State.

## 2. Materials and Methods

### 2.1. Study Design

This retrospective cross-sectional analysis was performed using deidentified antimicrobial resistance test results. The isolates included in the analysis were *E. coli* from urine specimens collected from outpatients in Washington State during a 7-year period. All antimicrobial resistance testing was performed by a single commercial reference laboratory.

### 2.2. Study Population

Outpatient urinary *E. coli* isolates from throughout the State of Washington were included in the analysis if they were tested at Quest Diagnostics from the years 2013 to 2019. Quest is a commercial laboratory that clinics, hospitals, and other healthcare facilities send specimens to for specialized laboratory testing services such as antimicrobial resistance testing. The first isolate from each individual in the dataset was used in the analysis. Any subsequent tests for that individual were excluded, as recommended by the Clinical and Laboratory Standards Institute for the creation of antibiograms [20].

### 2.3. Data Collection

Antibiotic resistance tests were performed at Quest Diagnostics’ laboratories in Seattle, WA, to determine the resistance of the isolates. VITEK* 2 (Biomérieux, Marcy-l’Étoile, France) methods delivered results for the genus and species for identification of the pathogen in the urine isolate and additionally provided the minimum inhibitory concentration (MIC) values for each antibiotic tested. Isolates were assigned “susceptible”, “intermediate”, and “resistant” interpretations based on MIC value breakpoints established by the CLSI Subcommittee on Antibiotic Susceptibility Testing [2]. Antibiotic resistance values were binarized for this analysis to be either “susceptible”, if categorized as susceptible using the MIC breakpoints, or “resistant” if categorized as resistant or intermediate using the MIC breakpoints [2].

Additional metadata included date tested, type of sample (e.g., urine, blood), patient age, and patient sex. This study assessed the results of resistance testing for five antibiotics, each representing a different class of antibiotics: ceftriaxone (cephalosporins), gentamicin (aminoglycosides), ampicillin (penicillins), ciprofloxacin (quinolones), and trimethoprim-sulfamethoxazole (sulfonamides). These antibiotics were selected in accordance with the Clinical Laboratory Standards Institute, the Infectious Diseases Society of America, and the European Society for Microbiology and Infectious diseases [2,4,20]. Through a research collaboration between Quest Diagnostics and the University of Washington, a deidentified dataset was supplied for analysis. This collaboration has been described previously [21].

### 2.4. Geographic Exposure Classification

Public Health Emergency Preparedness Regions (PHEPRs) have been created by the Washington State Department of Health to coordinate regional resources during times of public health need [22]. The nine Washington PHEPRs were used as a spatial exposure, since some counties had very few or no available isolate information. Participants were classified to a PHEPR by their home 3-digit zip code, whenever available, or by the zip code of the medical facility that ordered the antimicrobial resistance test.

### 2.5. Regression Analysis

Logistic regressions were performed in R (version 4.2.2) [23], with the Washington State PHEPRs as the predictors and susceptible or resistant as the binary outcomes. Robust standard errors and 95% confidence intervals with the adjusted prevalence odds ratio (aPOR) estimates for the fully adjusted regression for each antibiotic are presented. To calculate the adjusted prevalence odds ratios (aPOR) for antimicrobial resistance, sex (male and female binary), categorical age groups (0–18, 19–50, >50), and year of specimen collection (factor) were included in the model as covariates, selected a priori. The Central PHEPR was selected as the reference group because it had the largest number of isolates in the analysis and it contained Seattle, the largest metropolitan area in the state. Any isolates missing exposure, outcome, or covariate data were excluded from only the regression for which the missing data were needed.

### 2.6. Ethical Approval

The study protocols were approved by the Human Subjects Review Committee of the University of Washington (STUDY00008443).

## 3. Results

The Quest Diagnostics dataset included 56,022 *E. coli* urinary isolates, of which 15,157 were excluded due to not being the first *E. coli* isolate for the patient during the study period. Another 648 isolates were excluded due to missing data for age, sex, or county of residence. The final analyses included 40,217 *E. coli* isolates. (Table 1). The vast majority of isolates were from females (93.6%) and people over the age of 18 years old (90.6%). The year 2019 contained the most isolates, 8829, with over double the number of isolates from 2013, 4271. The last two years of data, 2018 and 2019, represented over 30% of the data.

The Central PHEPR, the reference group, had the most isolates (8097 isolates; 20.1%); the three regions (Central, North, and Southwest) with the most data collectively represented more than 50% of the isolates. Three other regions, collectively, represented fewer than 10% of the isolates: North Central, Northwest, and South Central. The North Central region was the smallest by far with 474 isolates and was the only PHEPR with fewer than 1000 isolates.

**Figure 1:**
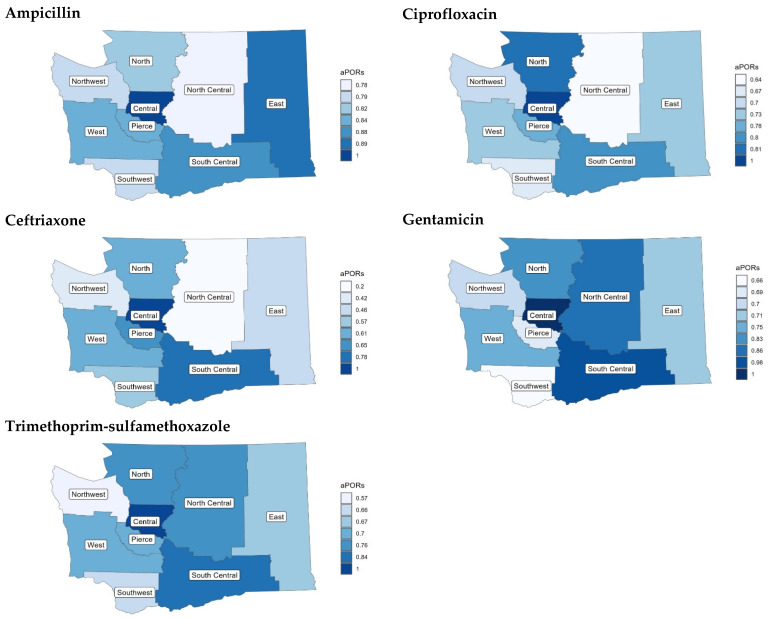
Map of Washington State divided into PHEPR and colored by adjusted prevalence odds ratio in relation to the reference region (Central) for the five antibiotics.

The overall resistance rates for each antibiotic varied greatly, ranging from 3.2% for ceftriaxone to 37.0% for ampicillin (Table 2). There were significant differences in aPORs for resistance between the Central PHEPR and other regions for all antibiotics, controlling for year of isolate collection, sex, and age group. *E. coli* antibiotic resistance was significantly less frequent in nearly all PHEPRs compared to the Central region, and no region had significantly higher resistance than the Central region, but the degree of differences varied by antibiotic (Figure 1). aPORs for antibiotic resistance were consistently 10–35% lower in all the non-Central regions for ampicillin, ciprofloxacin, and trimethoprim-sulfamethoxazole, and were also significantly lower for all other regions. Relative to the Central region, aPORs varied the most among regions for ceftriaxone (aPOR 0.20 to 0.78) and the least for ampicillin (aPOR 0.78 to 0.89). Only two regions did not have significantly different aPORs for one or more antibiotic: the South Central (not significant for ceftriaxone and gentamicin) and North Central (not significant for gentamicin). The South Central region aPOR was more similar to the Central region across all antibiotics; the Southwest and Northwest regions had some of most extreme aPORs, with greater differences in antimicrobial resistance compared to the Central region, across all the antibiotics.

## 4. Discussion

This study utilized urinary *E. coli* isolates from outpatients, primarily female, in Washington State, from 2013 to 2019. The five drugs selected for the analysis represent five different classes of antibiotics; resistance ranged from 3.2% for ceftriaxone to 37.0% for ampicillin. Our findings revealed substantial regional variations in the resistance patterns for all antibiotics tested. Compared to the Central PHEPR (containing Seattle), most other regions had significantly lower prevalence of antimicrobial resistance, and no region had significantly higher aPOR of resistance for any of the five antibiotics. Differences in resistance between the Central and eight other regions varied by antibiotic, with the variation being largest for ceftriaxone (low aPORs compared to the Central PHEPR) and smallest variation for ampicillin. These results reinforce the importance of using timely and local resistance data to create antibiograms and other tools to support antimicrobial stewardship, such as specific regional prescribing recommendations made by antimicrobial resistance stewards.

Provided these important insights into geographic variation in antimicrobial resistance, action can be taken to promote enhanced antibiograms to improve the accuracy of the data practitioners use for empiric prescribing. The large differences in antimicrobial resistance across Washington State suggest that prescribing patterns should be regionally specific, and that regionally specific antibiograms could help prevent further antimicrobial resistance and improve patient outcomes, when used appropriately by prescribers. This analysis of multisite data from a single commercial laboratory on outpatient urinary *E. coli* culture results provided further understanding of outpatient community-specific antimicrobial resistance patterns, as demonstrated at a large scale throughout the state. However, there are potentially more meaningful differences in smaller geographic communities than can be illuminated by the trends seen across the PHEPRs and this should be explored in further studies to determine the most useful and feasible spatial scale for which outpatient antibiograms could be tailored.

With over 40,000 isolates analyzed, this is one of the larger studies in the literature exploring antimicrobial resistance in likely community-acquired *E. coli* infections found in outpatient urinary isolates. The magnitude of the differences in resistance suggests that implementation of community-specific antibiograms could alter prescribing recommendations. While prescribing recommendations are more or less uniform [4,24], there should be additional emphasis to use the most regionally specific antibiogram available when making decisions for outpatient treatment.

A study utilizing data from the Global Burden of Diseases, Injuries, and Risk Factors Study found regional differences in the rates of morbidity and mortality caused by antimicrobial resistance to drugs [25]. Similarly, studies have described regional differences in antimicrobial resistance for pathogens within a country [26,27,28,29,30], and one discussed geographic antimicrobial resistance differences between the United States and Canada [28,31]. On a more granular scale, a study using Veterans’ Health Administration data found spatial clustering of outpatient *E. coli* antimicrobial resistance in the United States [32]. The findings of the present study are therefore consistent with those of previous studies. Furthermore, our findings support that those regional differences exist on even smaller geographic scales than previously examined and reiterate the suggestion that regional differences in antimicrobial resistance should be considered when planning programs and interventions to reduce antimicrobial resistance or improve patient treatment outcomes.

While few studies have explored reasons for regional antimicrobial resistance differences, an ecologic study from France found positive associations between extended spectrum beta-lactamase-producing *E. coli*, a common resistance mechanism, and several risk factors including percentage of people over 65 years of age, number of hospital beds, third-generation cephalosporin use, percentage of agriculture land, and poultry and pig density [33]. Some of these group-level associations could explain our finding of higher resistance in an urban center compared to more rural parts of the state. A study in Uganda found a significantly higher prevalence of multidrug resistance to outpatient *E. coli* and *Klebsiella* species in urban compared to the rural regions [26], and a study in Nepal similarly reported higher resistance prevalence closer to a major city [34]. On the other hand, a study in Germany found that the rural and urban differences were primarily inconsequential [35]. Mechanisms to account for such variation could be differences in prescribing and usage of antibiotics, population differences in susceptibility to infection, environmental persistence of antimicrobial resistance genes and antibiotic residues [36], and agricultural uses of antibiotics for crops and livestock [35,37]. These and other possible causes for variation should be further explored.

Antibiograms have been used extensively for antimicrobial stewardship, particularly in the hospital setting, as they provide pharmacies and physicians with information to prescribe empiric therapy, while waiting for culture and sensitivity results to come back [38]. While the utility of antibiograms is recognized, inpatient and outpatient resistance patterns can differ substantially [39,40]. Creating antibiograms based on outpatient data is challenging; not only does creating antibiograms require specialized microbiologic knowledge but additionally requires access to a centralized laboratory information system. Hospitals in a region may make their antibiograms available, yet these are not able to be generalized to other hospitals, regions, or outpatient infections [39,40]. Properly prescribing antibiotics not only helps with patient outcomes, but also prevents development of further resistance [38], and keeps costs lower, as resistant isolates are more expensive to treat [41].

The analysis was limited to grouping patients by PHEPR because of insufficient isolate sample size at a finer geographic level (such as county). The commercial laboratory does not have service points uniformly throughout the state, which has resulted in counties with no data or minimal data, and led to lower-powered analyses. The limited number of isolates from males does not allow the regional analysis to be stratified by sex, as might have been more appropriate to account for potential antimicrobial resistance differences between the sexes [21]. There should be exploration of further covariates in regions that could be predictive of differences in antimicrobial resistance, such as rurality, antibiotic usage on nearby farms, antibiotics sprayed in orchards, hospital bed density, or other regionally specific data that could further understanding of regional differences in resistance. Additional data from 2020 to 2024 were not included in this analysis due to SARS-CoV-2 pandemic likely skewing the data starting in 2020. While we did not explore this in our paper, researchers should investigate how pandemics and interruptions in typical access to care possibly skew antibiograms.

By only using the first isolate per unique individual, recurrent or unresolved infections in the data source were minimized. Although we used only the first isolate from each patient, it is likely that some people with previous *E. coli* infections were still included in the analysis because they had been tested at other laboratories or the prior infection occurred before the study window. Using only the first specimen collected during 2013–2019 may have biased the results to appear as if there is less antimicrobial resistance, because previous treatment with an antibiotic is associated with increased antimicrobial resistance [42,43,44]. Therefore, our results may underestimate overall community antimicrobial resistance, and specifically antimicrobial resistance for subpopulations with recurrent infections [42,43,44].

Our study did not account for the medical risk factors and comorbidities for people who accessed care through an outpatient center utilizing a commercial laboratory compared to those who have access to a medical facility with a laboratory capacity in-house, resulting in potential selection bias. Additional selection bias could potentially be present due to Quest Diagnostics being one of several commercial companies in the region and the presence of in-house laboratories so we cannot assume this is a completely representative sample of the state outpatient *E. coli* isolates; however, we have over 40,000 isolates included in the analysis, which is large compared to similar literature. This study used only samples from outpatient tests, but this does not eliminate the possibility that people could have been recently discharged from the hospital and been included in the dataset. Using outpatient samples only, this study cannot be generalized to the inpatient population and their antibiograms.

While there were limitations in this analysis, this study included a substantial number of outpatient isolates from the entirety of Washington State as serviced by a commercial laboratory, which allowed for the comparison of regions within politically meaningful boundaries. The findings suggest that we should be tailoring antibiograms to be more regionally specific to best support treatment of patients and to reduce the emergence of antimicrobial resistance in clinically important outpatient antibiotics. Further studies are needed to (1) better understand which levels of regional classification (e.g., neighborhood, county, region of state) are the most meaningful when creating community-specific antibiograms and (2) delve into the different drivers behind resistance, so that distal drivers of emergence can be identified and used to prevent further antimicrobial resistance. While providing more accurate antibiograms for providers is important, larger systemic changes, such as reevaluating the antibiotic use of multiple industries, will be necessary to mitigate further resistance.

## Figures and Tables

**Table 1:** Demographics of outpatients with urinary *E. coli* isolates, Washington State, 2013–2019 (N = 40,217).

	Number	%
PHEPR ^a^		
Central	8097	20.1
East	2783	6.9
North	7451	18.5
North Central	474	1.2
Northwest	1300	3.2
Pierce	5962	14.8
South Central	1189	3.0
Southwest	7857	19.5
West	5104	12.7
Year of collection		
2013	4271	10.6
2014	3499	8.7
2015	4086	10.2
2016	4313	10.7
2017	7656	19.0
2018	7563	18.8
2019	8829	22.0
Sex		
Male	2589	6.4
Female	37,628	93.6
Age group, years		
0–18	3781	9.4
19–50	18,326	45.6
>50	18,110	45.0

^a^ Public Health Emergency Preparedness Regions (PHEPRs) of Washington State. No covariates were missing more than 5% of the data.

**Table 2:** Adjusted prevalence odds ratios for antibiotic resistance among outpatient *E. coli* isolates in Washington State, 2013–2019.

			Ampicillin (n = 40,042)	Ciprofloxacin (n = 40,214)	Ceftriaxone (n = 40,017)	Gentamicin (n = 40,217)	Trimethoprim- Sulfamethoxazole (n = 40,170)
Overall resistance			37%	10%	3%	5%	18%
	n	(%)	adjusted POR ^b^ (95% CI)				
PHEPR ^a^							
Central	8097	20.1	Ref.	Ref.	Ref.	Ref.	Ref.
East	2783	6.9	0.89 (0.81–0.98)	0.73 (0.62–0.85)	0.46 (0.35–0.60)	0.71 (0.57–0.88)	0.67 (0.59–0.75)
North	7451	18.5	0.82 (0.76–0.88)	0.81 (0.72–0.90)	0.61 (0.51–0.72)	0.83 (0.72–0.97)	0.76 (0.70–0.83)
North Central	474	1.2	0.78 (0.64–0.95)	0.64 (0.46–0.90)	0.20 (0.06–0.63)	0.86 (0.56–1.31)	0.76 (0.59–0.97)
Northwest	1300	3.2	0.79 (0.70–0.90)	0.70 (0.57–0.86)	0.42 (0.26–0.65)	0.70 (0.53–0.93)	0.57 (0.48–0.67)
Pierce	5962	14.8	0.84 (0.78–0.90)	0.78 (0.70–0.87)	0.65 (0.53–0.78)	0.69 (0.59–0.81)	0.70 (0.64–0.76)
South Central	1189	3.0	0.88 (0.77–1.00 ^c^)	0.80 (0.65–0.99)	0.78 (0.57–1.08)	0.98 (0.75–1.28)	0.84 (0.72–0.99)
Southwest	7857	19.5	0.79 (0.74–0.84)	0.67 (0.60–0.75)	0.57 (0.48–0.68)	0.66 (0.57–0.77)	0.66 (0.61–0.72)
West	5104	12.7	0.84 (0.78–0.91)	0.73 (0.65–0.82)	0.61 (0.49–0.75)	0.75 (0.64–0.89)	0.70 (0.64–0.76)

^a^ Public Health Emergency Preparedness Regions (PHEPRs) of Washington State. ^b^ Adjusted prevalence odds ratios (aPORs) are adjusted for year of isolate collection, sex of the patient, and age group of the patient. ^c^ This confidence interval does not include 1.00 but, due to rounding, 1.00 is the upper limit for the table.

## Data Availability

The datasets presented in this article are not readily available because of data sharing policies of the data source.
